# Biomimicking Bone–Implant Interface Facilitates the Bioadaption of a New Degradable Magnesium Alloy to the Bone Tissue Microenvironment

**DOI:** 10.1002/advs.202102035

**Published:** 2021-10-28

**Authors:** Wenting Li, Wei Qiao, Xiao Liu, Dong Bian, Danni Shen, Yufeng Zheng, Jun Wu, Kenny Y. H. Kwan, Tak Man Wong, Kenneth M. C. Cheung, Kelvin W. K. Yeung

**Affiliations:** ^1^ School of Materials Science and Engineering Peking University Beijing 100871 China; ^2^ Department of Orthopedics and Traumatology The University of Hong Kong Pokfulam Hong Kong China; ^3^ Shenzhen Key Laboratory for Innovative Technology in Orthopaedic Trauma Department of Orthopaedics and Traumatology The University of Hong Kong‐Shenzhen Hospital Shenzhen 518053 China; ^4^ Department of Orthopedics Guangdong Provincial People's Hospital Guangdong Academy of Medical Sciences Guangzhou 510080 China

**Keywords:** bone–implant interfaces, immunomodulation, magnesium alloy, osteogenesis, osteointegration

## Abstract

The most critical factor determining the success of biodegradable bone implants is the host tissue response, which greatly depends on their degradation behaviors. Here, a new magnesium‐based implant, namely magnesium–silicon–calcium (Mg–0.2Si–1.0Ca) alloy, that coordinates its biodegradation along with the bone regenerative process via a self‐assembled, multilayered bone–implant interface is designed. At first, its rapid biocorrosion contributes to a burst release of Mg^2+^, leading to a pro‐osteogenic immune microenvironment in bone. Meanwhile, with the simultaneous intervention of Ca and Si in the secondary phases of the new alloy, a hierarchical layered calcified matrix is rapidly formed at the degrading interface that favored the subsequent bone mineralization. In contrast, pure Mg or Mg–0.2Si alloy without the development of this interface at the beginning will unavoidably induce detrimental bone loss. Hence, it is believed this biomimicking interface justifies its bioadaptability in which it can modulate its degradation in vivo and accelerate bone mineralization.

## Graphical abstract

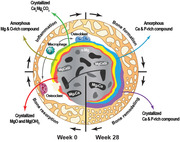

## 1 Introduction

The substantial burden of disability and suffering caused by an increasing number of musculoskeletal injuries has become a significant problem in public health and social welfare in recent years.^[^ ^1^ ^]^ Although conventional metallic biomaterials, such as stainless steel and titanium, have been utilized to design orthopedic devices to facilitate bone healing, there remain some problems impeding the clinical usage of these materials. For instance, bone loss or osteoporosis could occur around these implants because of a “stress shielding effect,” meaning the transferring of load to bone can be hindered as a result of the higher elastic modulus of implants compared with bone tissue.^[^ ^2^ ^]^ Moreover, the implant remaining in the fractured site after complete healing of bone can cause discomfort to patients. Especially for such groups as children, teenagers, and athletes, the implants usually should be removed via a secondary operation, which may result in complications and a burden for both surgeons and patients.^[^ ^3^ ^]^ Biodegradable magnesium (Mg), with its superior biodegradability and similar elastic modulus and density to bone, has attracted much attention in recent years.^[^ ^4^ ^]^ A biodegradable Mg‐based implant can gradually degrade along with the healing of bone, allowing secondary surgery for implant removal to be prevented. Besides, as a crucial element in the bone matrix and a second messenger in intracellular signaling,^[^ ^5^ ^]^ released magnesium from the implant has been found to contribute to new bone formation in various ways.^[^ ^6^, ^7^, ^8^ ^]^ However, due to the highly dynamic degradation process of various Mg‐based implants and the distinct host response elicited at different stages of bone healing, previous studies concerning the in vivo performance of Mg‐based implants remain highly controversial.

The host tissue response is critical when it comes to the success of biodegradable implants.^[^ ^9^ ^]^ Although Mg‐based implants have been extensively studied as orthopedic implants, their fast degradation rate and hydrogen gas production have been associated with the failure of tissue repair.^[^ ^10^, ^11^ ^]^ In addition, the rapid release of Mg^2+^ was reported to compromise bone mineral density due to the suppression of cell viability and mineral deposition.^[^ ^6^, ^12^ ^]^ Therefore, it is important to adapt the degradation kinetics of Mg‐based implants to comply with the biological process of bone healing in order to maintain a pro‐osteogenic peri‐implant tissue microenvironment. It has been observed that the formation of biocompatible calcium phosphate layers between bone tissues and the degrading implants is the key to coordinating the degradation behavior of biodegradable implants to the subsequent bone integration process.^[^ ^7^, ^13^, ^14^ ^]^ Despite numerous attempts to control the degradation of Mg implants with various surface coating techniques, surface cracking and pitting corrosion that may lead to the penetration of corrosive body fluid and uncontrolled degradation have been quite common after implantation.^[^ ^15^, ^16^ ^]^ Instead, the dense and uniform degradation products spontaneously formed at the bone–implant interface have been shown to be superior in controlling the biodegradation of the metallic implants and facilitating the adaption of the implant to the host tissues.^[^ ^13^, ^17^ ^]^ Indeed, calcium phosphate enriched degradation products not only assist the integration of Mg implants with host bone,^[^ ^7^, ^13^ ^]^ but also regulate the peri‐implant tissue microenvironment to promote new bone formation.^[^ ^7^, ^8^, ^18^ ^]^

Calcium (Ca) and silicon (Si) are two of the most popular elements for the alloying of Mg‐based implants. The addition of Si in Mg can increase the fluidity of the melt during the smelting process of the alloys and the formation of Mg_2_Si phase caused by the low solubility of Si in Mg (0.006 wt%) can strengthen the alloys due to the second phase hardening effect.^[^ ^19^, ^20^ ^]^ However, the ductility of the Mg–Si alloy could be compromised by the presence of Mg_2_Si particles.^[^ ^20^ ^]^ Therefore, elements like Ca were added in order to refine the microstructure of the alloy by modifying the coarse Mg_2_Si phase into a fine form.^[^ ^21^ ^]^ Meanwhile, both Ca and Si are fundamental elements that contribute to the formation of a degradation layer at the bone–implant interface. Ca is the major component of the calcium phosphate enriched degradation products that contribute to osseointegration and new bone formation.^[^ ^13^ ^]^ Si is able to increase the solution stability of amorphous calcium phosphate and drive the amorphous calcium phosphate to its mineralized state.^[^ ^22^ ^]^ Moreover, both Ca and Si present as the essential elements in the bone formation process. For instance, Si is beneficial to the maintenance of bone mineral density and connective tissue function.^[^ ^23^ ^]^ In addition, it not only promotes the proliferation and differentiation of osteoblasts, collagen secretion, and bone matrix mineralization, but it also inhibits bone resorption.^[^ ^23^, ^24^ ^]^ In addition, Ca has been recognized as a major component of bone tissue and a vital element in maintaining bone microstructure.^[^ ^25^ ^]^ The recruitment, viability, and differentiation of osteoblasts and osteoclasts are mainly regulated by Ca^2+^ through the transmembrane calcium‐sensing receptor (CaSR).^[^ ^26^ ^]^ Hence, the incorporation of Si and Ca into the Mg matrix may regulate the biodegradability of Mg‐based alloys and improve their bioadaptability.

In this study, we designed a new generation of Mg–*x*Si–*y*Ca alloys with improved mechanical and biodegradation properties to regulate the bioadaption of this degradable alloy to host bony tissue. We demonstrated that simultaneous incorporation of Si and Ca, rather than Si alone, convinced superior new bone formation adjacent to the implant. To better understand the relationship between implant degradation and bone regeneration processes, we explored the dynamic changes of the implant‐to‐bone interface at the nanoscale level in vivo. The results demonstrated that the Mg–Si–Ca implant could coordinate its biodegradation and in situ osteogenesis through a self‐assembled, multilayered implant–tissue interface (i.e., a crystalline MgO and Mg(OH)_2_ layer next to the implant, a layer of amorphous Mg‐ and O‐rich compound followed by a layer of amorphous Ca‐ and P‐rich compound, and a layer of crystalline calcium phosphate integrated with mineralized bone). Moreover, the biomimicking bone–implant interface triggered by the Mg–Si–Ca alloy tailored the release kinetics of Mg, Si, and Ca ions, leading to the establishment of a pro‐osteogenic tissue microenvironment in the peri‐implant area.

## 2 Results

### 2.1 Microstructure Analysis

**Figure 1:**
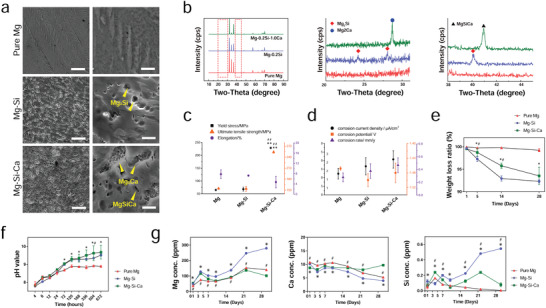
Characterization of Mg, Mg–Si, and Mg–Si–Ca alloy. a) Microstructure of pure Mg, Mg–0.2Si, and Mg–0.2Si–1.0Ca alloy by ESEM (scale bar = 30 µm), as well as higher magnification of the microstructure (scale bar = 3 µm) showing intermetallic phases. b) XRD spectra of pure Mg, Mg–0.2Si, and Mg–0.2Si–1.0Ca alloy. c) Yield strength, ultimate tensile strength, and elongation of pure Mg and Mg–0.2Si–1.0Ca alloy. d) Corrosion current density, corrosion potential, and corrosion rate of pure Mg and Mg–0.2Si–1.0Ca alloys based on the results of electrochemical tests. e) The weight loss ratio of pure Mg and Mg–0.2Si–1.0Ca alloy during 28 day incubation in Hank's solution. f) The changes in pH value of Hank's solution incubating pure Mg and Mg–0.2Si–1.0Ca alloy during a 28 day period. g) The concentration of Mg, Ca, and Si in the DMEM medium during 30 day incubation with pure Mg or Mg–0.2Si–1.0Ca alloy under physiological condition. (Data are shown as mean ± s.d., *n* = 3 per group. * or # represent *p* < 0.05 compared with pure Mg or Mg–Si group, respectively, by one‐way ANOVA with Tukey's post hoc test.)

The microstructure of extruded Mg‐(0.2, 0.4, 1.0)Si alloy and Mg–*x*(*x* = 0.2, 0.4, 1.0)Si–*y*(*y* = 0.5, 1.0)Ca alloys under scanning electron microscopy (SEM) (**Figure** **1**a; and Figure S1a,b, Supporting Information) showed the presence of second phases in Mg matrix. Using X‐ray diffraction (XRD) analysis, the intermetallic phase in Mg–*x*Si was detected to be Mg_2_Si, while the intermetallic phases in Mg–*x*Si–*y*Ca alloys were detected to be Mg_2_Si, Mg_2_Ca, and MgSiCa (Figure 1b; and Figure S1c, Supporting Information). To be specific, the Mg_2_Ca and MgSiCa phases were identified in Mg–(0.2, 0.4)Si–(0.5, 1.0)Ca alloys. Mg_2_Ca presented in polygonal forms and MgSiCa in its fine needle‐like shape or particle shape. In the Mg–1.0Si–(0.5, 1.0)Ca alloys, both the Mg_2_Si and MgSiCa phases were identified. Mg_2_Si was in particle form and MgSiCa in polygonal form.

### 2.2 In Vitro Degradation Behavior of the Alloys in Hank's Solution

The in vitro degradation test for detecting the sequence of the degradation rate of the alloys was carried out in Hank's solution. With the increasing content of Si, the weight loss ratio of Mg–*x*Si and Mg–*x*Si–*y*Ca alloys (Figure 1e; and Figure S1d,e, Supporting Information), as well as the pH value of the immersion solution (Figure 1f; and Figure S1d,e, Supporting Information) gradually increased at every designated time point. The corrosion current density and corrosion rate of Mg–*x*Si and Mg–*x*Si–*y*Ca alloys based on electrochemical tests also increased with the rise in Si content (Figure S2a,b, Supporting Information). Collectively, our results suggest that the addition of Si into the Mg matrix can accelerate the degradation rate. Among the Mg–*x*Si–*y*Ca alloys, Mg–1.0Si–(0.5, 1.0)Ca alloy showed the fastest degradation rate with more than 50% weight loss after 5 days of immersion. Interestingly, the presence of Ca tended to decelerate the degradation; according to the immersion test, the Mg–*x*Si–1.0Ca alloy had a lower degradation rate compared with its corresponding Mg–*x*Si–0.5Ca alloy (Figure S1d,e, Supporting Information).

### 2.3 Cytocompatibility Tests of the Alloys

The cell viability results using hMSC indicated that the cytocompatibility of Mg–(0.2, 0.4, 1.0)Si alloys and Mg–*x*(*x* = 0.2, 0.4, 1.0)Si–y(y = 0.5, 1.0)Ca alloys was acceptable according to ISO 10993‐5 standard. Indeed, Mg–0.2Si extract significantly promoted the cell proliferation after 3‐ and 5‐days culture (Figure S3a, Supporting Information). Meanwhile, The Mg–0.2Si–1.0Ca alloy showed the best cytocompatibility among all the tested Mg–*x*Si–*y*Ca alloys, as the viability of hMSC cultured with 100% extract of Mg–0.2Si–1.0Ca alloy was significantly higher than those cultured with 100% extract of any other Mg–*x*Si–*y*Ca alloys at day 3 and 5. The cell viability results using MC3T3 indicated that the extract of Mg–0.2Si showed the highest cytocompatibility, while the extract of Mg–1.0Si toxified the cells. For Mg–*x*(*x* = 0.2, 0.4, 1.0)Si–*y*(*y* = 0.5, 1.0)Ca alloys, only the 100% extract of Mg–0.2Si–0.5Ca failed to maintain 80% cell viability at day 1, all undiluted extracts from Mg–*x*Si–*y*Ca alloys led to significantly lower cell viability than 70% of the control at days 3 and 5. However, Mg–0.2Si–1.0Ca remained the best among the tested Mg–*x*Si–*y*Ca alloys in terms of cytocompatibility, manifested by the highest cell viability determined at days 3 and 5.

### 2.4 Mechanical Properties of Mg–*x*Si–*y*Ca Alloys

Since Mg–0.2Si and Mg–0.2Si–1.0Ca alloys were the optimal selections among their other counterparts in terms of in vitro degradation rate, mechanical properties, and cytocompatibility, they were therefore selected for further in vitro and in vivo studies. Pure Mg prepared by the extruded method served as a control (Figure 1a–g). No significant difference was observed between P–Mg and Mg–Si for their yield strength (YS), ultimate tensile strength (UTS), and elongation. However, the yield strength of Mg–Si–Ca was four times higher than that of P–Mg and Mg–Si alloy (Figure 1c). With the presence of Mg_2_Ca and MgSiCa phases (Figure 1a,b), the Mg–Si–Ca alloy showed a significantly higher YS at ≈225 MPa and UTS at ≈255 MPa (Figure 1c), making it comparable to the mechanical strength of a commercialized Mg–Ca–Zn alloy implant.^[^ ^13^ ^]^ In contrast, pure Mg and Mg–Si alloy only had YS at ≈70 MPa and UTS at ≈150 MPa. There was no significant difference in terms of the elongation of pure Mg, Mg–Si alloy, and Mg–Si–Ca alloy.

### 2.5 Ion Release Profile under Physiological Condition In Vitro

Interestingly, although there was no significant difference in the corrosion current density, corrosion potential, and corrosion rate among pure Mg, Mg–Si alloy, and Mg–Si–Ca alloy (Figure 1d), the weight loss (Figure 1e) and changes of pH value (Figure 1f) in the 28‐day Hank's solution immersion test showed that the degradation of Mg–Si and Mg–Si–Ca alloy was faster than that of pure Mg. The accumulated magnesium ion concentration measured in the routinely refreshed Dulbecco's modified Eagle's medium (DMEM) confirmed the Mg–Si alloy to be the most quickly degraded one among the other tested groups (Figure 1g). More importantly, the ion release profile of pure Mg and Mg–Si–Ca in the initial period and the end of the 30‐day immersion test seemed distinct from each other (Figure 1g). The release of Mg ions from Mg–Si–Ca alloy was faster than that from pure Mg in the first week; however, this tendency reversed in the last week of the immersion test. Meanwhile, the Ca concentration in the pure Mg immersed media, which was higher at the beginning, became lower than that of the Mg–Si–Ca alloy. The Si concentration in the Mg–Si and Mg–Si–Ca alloys immersed media was higher than the medium collected from the pure Mg group, possibly due to the degradation of the Mg_2_Si and MgSiCa phase.

### 2.6 Bone Regeneration and Osseointegration

**Figure 2:**
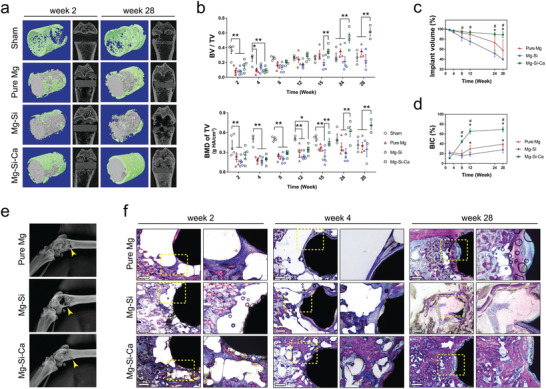
The degradation of the implants and osseointegration. a) Representative micro‐CT images and 3D reconstruction of the implant (gray in color) and bone around the implant (green in color) at week 2 and week 28 postoperation. b) Micro‐CT measurements of BV/TV and BMD of TV during the 28‐week implantation period. *n* = 5, *p < 0.05 and **p < 0.01 by two‐way ANOVA with Tukey's post hoc test. c,d) Micro‐CT measurements showing the change in c) implant volume and d) bone–implant contact (BIC) during the 28‐week implantation period. *n* = 5, * or # represent *p* < 0.05 compared with pure Mg or Mg–Si group, respectively, by two‐way ANOVA with Tukey's post hoc test. e) Representative radiographs of femur with the implants at week 28 postoperation. f) Representative Giemsa staining showing osseointegration and new bone formation around the implants at week 2, 4, and 28 postoperation (scale bar = 500 µm).

The **in vivo** degradation and osteogenic potential of the alloys in rat femora were sequentially evaluated using live animal microcomputed tomography (CT). Representative micro‐CT images and 3D reconstructions demonstrated that the bone volume surrounding the Mg–Si–Ca implant was higher than that of the pure Mg group (**Figure** **2**a; and Figure S4b, Supporting Information). The lowest amount of bone was found in Mg–Si group, in which a large hollow area was observed around the implant, resulting in an even lower bone volume than the sham group (Figure 2a). This is confirmed by the corresponding quantification of high‐density bone volume to total bone tissue volume ratio (BV/TV) and the bone mineral density of the total bone tissue volume (BMD of TV) around the implant (Figure 2b). The bone volume around the Mg–Si–Ca alloy continuously increased over the 28‐week implantation, while the bone volume of the Mg–Si group did not change significantly after the operation. At week 28, the BV/TV around the Mg–Si–Ca implant was tripled as compared with the Mg–Si group and doubled compared with the pure Mg group. Moreover, it was evident from micro‐CT data that the decrease in implant volume of Mg–Si alloy was significantly faster than that of pure Mg and Mg–Si–Ca alloy (Figure 2c). Consequently, the residual Mg–Si–Ca implant was higher than 80% at week 28, while the residual pure Mg and Mg–Si alloy was only around 60% and 40%, respectively. Sequential X‐ray images of the rat femora over the 28 weeks after the operation showed that the surgical wound gradually healed after the placement of the Mg–Si–Ca implant (Figure 2e; and Figure S4a, Supporting Information). In contrast, an obvious dark area appeared on the implantation site of either pure Mg or Mg–Si alloy, indicating that these two degradable metals compromised the bone healing process.

**Figure 3:**
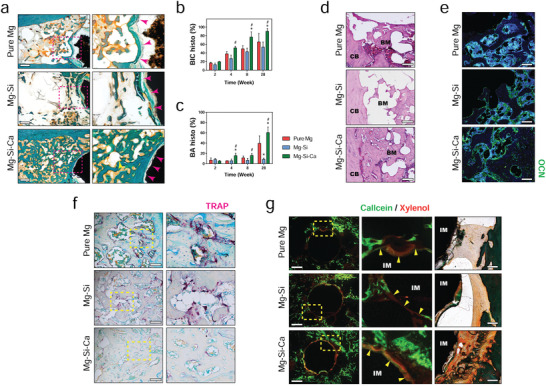
New bone formation triggered by the implants. a) Representative Goldner's trichrome staining showing new bone formation around the implants at week 28 postoperation (scale bar = 500 µm). b,c) Histological measurement of BIC b) and bone area (BA) surrounding the implants c) at week 2, 4, 8, and 28 postoperation. *n* = 3, * or # represent *p* < 0.05 compared with pure Mg or Mg–Si group, respectively, by two‐way ANOVA with Tukey's post hoc test. d) Representative H&E staining showing new bone formation around the implants at week 28 postoperation (scale bar = 200 µm). CB: cortical bone; BM: bone marrow. e) Representative immunofluorescent staining showing the expression of OCN around the implant at week 28 postoperation (scale bar = 100 µm). f) Representative TRAP staining showing osteoclastic activity around the implant at week 28 postoperation (scale bar = 200 µm). g) Representative images of calcein/xylenol labeling (Left, scale bar = 500 µm) with correspond Goldner's trichrome staining images (right, scale bar = 100 µm) showing bone regeneration around the implants at week 8 postoperation. IM: implant; BM: bone marrow.

Our micro‐CT data also revealed a significantly higher bone–implant contact (BIC) ratio in the Mg–Si–Ca group than in the pure Mg or Mg–Si group starting from week 8 after the operation (Figure 2d). This was further verified by Giemsa staining on undecalcified bone sections at postoperative weeks 2, 4, and 28 (Figure 2f). The cavities that formed around the pure Mg and Mg–Si–Ca implants during the early period after implantation were gradually replaced by newly formed bone tissues (Figure 2f). However, the cavities around the Mg–Si implant remained unhealed even after 28 weeks of postoperation. The increase in the amount and quality of bone tissue surrounding the Mg–Si–Ca implant was confirmed by Goldner's trichrome staining on undecalcified bone sections (**Figure** **3**a) and hematoxylin and eosin (H&E) staining (Figure 3d) on decalcified bone sections at week 28 after the operation. Quantitative measurement of BIC and bone area (BA) based on the histology data showed that the osseointegration and new bone formation in the Mg–Si–Ca group were superior to those in the pure Mg or Mg–Si group (Figure 3b,c). Furthermore, using immunofluorescent staining, we found that the osteogenic activity, manifested by the expression of osteocalcin (OCN), was significantly higher in the femurs with Mg–Si–Ca implants than in the femurs with pure Mg or Mg–Si implants (Figure 3e). Meanwhile, tartrate‐resistant acid phosphatase (TRAP) staining showed that osteoclastic activity in the Mg–Si–Ca group was significantly lower than in the pure Mg group or Mg–Si group at week 28 posstoperation (Figure 3f).

To understand the relationship between the degradation behavior and the osteogenic effects of the implants, we distinguished the newly formed bone from the old bone using fluorescent labeling. As demonstrated in the fluorescent images (Figure 3g), the new bone formation around the Mg–Si–Ca implant was more aggressive as compared with that of pure Mg implants and Mg–Si implants. Moreover, a significantly increased calcium deposition could be observed on the surface of the Mg–Si–Ca implant by fluorescence. This was confirmed by corresponding Goldner's trichrome staining, showing a more unified calcium‐based layer and more mineralized bone tissues formed at the bone–implant interface of Mg–Si–Ca implants when compared with pure Mg an Mg–Si implants (Figure 3g).

### 2.7 Mechanical Strength of Newly Formed Bone

Since the quality of bone is not only influenced by the architecture and connectivity of trabeculae but also affected by its mechanical properties at the molecular level, we further determined the mechanical properties of the newly formed bone around pure Mg and Mg–Si–Ca implants via a nanoindentation test. The modulus and hardness were relatively higher near the implant (≈50–100 µm from the edge of the implant) and dropped at ≈150 µm from the implants (Figure S5a–d, Supporting Information). The hardness of bone around pure Mg and Mg–Si–Ca implants were both ≈0.7 GPa, which was similar to the hardness in the sham group (Figure S5f, Supporting Information). Meanwhile, the elastic modulus of bone adjacent to the pure Mg and Mg–Si–Ca implants were ≈17 and 22 GPa, respectively, and these values were both significantly higher than that in the sham group (Figure S5e, Supporting Information).

### 2.8 In Vivo Degradation Behavior

**Figure 4:**
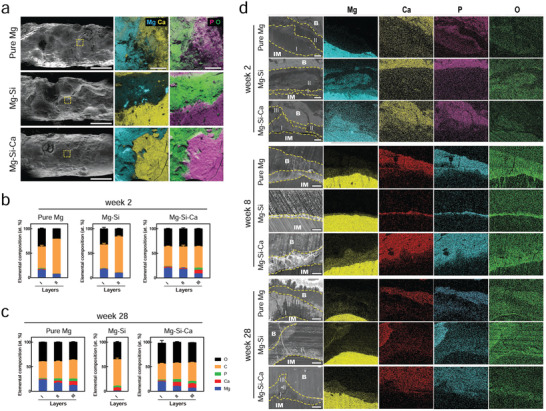
Degradation behavior of implants. a) Surface morphology (scale bar = 1 mm) and element distribution of the implants retrieved from the femora 12 weeks after implantation. b,c) Chemical composition of multilayered degradation products on implants at week 2 b) and week 28 c) after implantation determined using SEM‐EDX. These data are the mean ± s.d., *n* = 5. d) Cross sectional SEM images and corresponding EDX mapping showing the bone–implant interface at week 2, week 8, and week 24 postoperation (scale bar = 50 µm), dotted line refers to the margin of implant, bone, and degradation products observed under SEM.

Since we observed significant differences in the degradation rate of the three kinds of implants in vivo (Figure 2c), which echoed our in vitro finding on the different degradation behaviors of the implants in culture medium (Figure 1g), we retrieved the implants from rat femora at 12 weeks after the operation and studied the degradation products using energy dispersive X‐ray spectroscopy (SEM–EDX ) (**Figure** **4**a). There were significantly more concavity and pits found on pure Mg and Mg–Si alloy rather than Mg–Si–Ca alloy. At least two distinct layers were characterized by EDX mapping on the implants. The layer rich in Ca and phosphorus (P) on the surface was thicker in the Mg–Si–Ca group than it was in the pure Mg group and Mg–Si group. The layer underneath, which was exposed because of the destruction of the surface layer during the extraction, was rich in Mg and O. Using SEM‐EDX analysis, we further demonstrated the bone–implant interface of the three Mg‐based implants at postoperative weeks 2, 8, and 28 on a microlevel (Figure 4b–d). At week 2, compositional analysis demonstrated an Mg‐enriched zone in the immediate vicinity of the implant surface. As the main degradation product at this stage, this layer was incorporated with C and O, as well as a small amount of Ca and P (Figure 4b,d). Meanwhile, another Ca‐ and P‐containing zone, with lower Mg content, could be found in the outer layer of the degradation product. In the Mg–Si–Ca alloy, an additional Ca‐ and P‐enriched layer, with a similar chemical composition to the surrounding bone, could be identified (Figure 4b,d). At week 8, with the thickening of the degradation product, the O content in the inner layer of the interface, as well as the Ca and P contents in the outer layer of the interface, significantly increased in both the pure Mg and Mg–Si–Ca groups (Figure 4d). However, the degrading interface in Mg–Si alloy became even thinner, despite an increase in the Ca and P content. At week 28 after implantation, with a continuous increase in Ca and P content in the bone–implant interface, a bone‐like degradation layer where new bone integrated with could be identified in both the pure Mg and Mg–Si–Ca groups (Figure 4c,d). In contrast, a well‐organized degrading interface was not seen on Mg–Si alloy.

**Figure 5:**
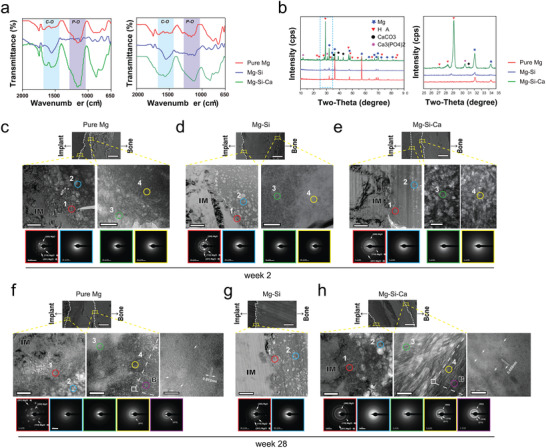
Changes in the bone–implant interface after implantation a) FTIR spectra of the degradation products on implants at week 8 and week 24 after implantation. b) X‐ray diffraction patterns of the implants retrieved from the femora 12 weeks after implantation. c–h) Representative TEM images showing the nanostructure of key regions of the bone–implant interface at week 2 c–e) and week 28 f–h) after implantation (scale bar = 200 nm). SEM images in the upper line indicate the location of key regions at the interface (scale bar = 30 µm), dotted line refers to the margin of implant, bone and degradation products. Corresponding SAED patterns of different layers of degradation products are shown in the lower line. IM: implant; B: bone. Nanostructure of the interface adjacent to integrated bone is shown at high magnification (scale bar = 10 nm).

To investigate the dynamic changes in the chemical composition of degradation products of the implants under the in vivo scenario, we examined the bone–implant interface on the cross‐sections of the implants. Fourier transform infrared (FTIR) spectra of the interface showed that the O—C—O stretch bands at around 1400 and 840 cm^−1^, as well as the composed and overlapped bands in the 960–1200 cm^−1^ range resulting from P—O stretching vibration, were stronger in the Mg–Si–Ca group rather than in pure Mg and Mg–Si alloy (**Figure** **5**a). Further analysis using XRD suggested that the presence of CaCO_3_, Ca_3_(PO_4_)_2_ and HA was more evident in the surface degradation products of Mg–Si–Ca implants (Figure 5b).

### 2.9 Bone–Implant Interface in Nanoscale

As we have characterized the multilayer structure of the bone–implant interface of the degrading implants using SEM‐EDS, the nanoscale structure and crystallographic characterizations of the representative regions in the interface were further investigated by transmission electron microscopy (TEM; Figure 5c–h). At week 2, the first layer of the degradation product (Region 1; Figure 5c–e; and Figure S6a, Supporting Information), which is formed on the surface of the three kinds of implants, was found to contain a lot more Mg and O than the other elements (Figure S6a, Supporting Information). This initial layer of degradation product, with a thickness of only ≈100 nm, was confirmed to be crystalline MgO and Mg(OH)_2_ by selected area electron diffraction (SAED). The amorphous structure (Region 2) located next to the crystalline Mg and O compound layer was found to consist of less Mg–O and more Ca–P. Meanwhile, TEM observation concerning the interface between the Mg‐rich layer (Region 3) and the Ca‐ and P‐rich layer (Region 4) close to it revealed that, with the growing distance from the implant surface, the Mg content decreased dramatically, while Ca, P, and C increased continuously (Figure S6a, Supporting Information). Moreover, in these three implants, both Regions 3 and 4 showed amorphous ring patterns by SAED (Figure 4a). Nevertheless, in the Mg–Si–Ca alloy group, the amorphous structure in Region 4 contained higher amounts of Ca and P than was observed in the pure Mg group, making layer III distinguishable by SEM‐EDX. In contrast, more C in Region 4 were significantly found on the degradation products of Mg–Si implant, suggesting that it failed to formulate the osseointegration upon biodegradation.

As the implantation time reached 28 weeks, TEM analysis of the bone–implant interface demonstrated that the crystalline MgO and Mg(OH)_2_ layer was still found right next to these three materials (Region 1; Figure 5f–h). There was no significant change in the composition of this region (Figure S6b, Supporting Information). The involvement of Ca and P in the amorphous Mg and O compounds (Region 2) was only detected in the Mg–Si–Ca group. The major difference between the bone–implant interface results at week 2 and week 28 was found in the Ca‐ and P‐enriched layer connecting the surrounding bone. Given a significantly increased amount of Ca and P in this layer compared with that at week 2, the Ca and P content in Region 3 of pure Mg was still lower than the corresponding area in the Mg–Si–Ca group (Figure S6b, Supporting Information). Moreover, a gradual change from an amorphous structure in Region 3 to a crystalline structure in Region 4 was evidenced by the presence of (211) and (002) planes in SAED in both the pure Mg and Mg–Si–Ca groups (Figure 5f,h). Since the diffraction pattern from Region 4 was identical to that from the adjacent bone, we further demonstrated that the nanostructure of this area was like that of typical bone, exhibiting mineralized calcium phosphate, which has a bone‐specific *c*‐axis orientation (Figure 5f,h).

### 2.10 Formation of Degradation Layer In Vitro

**Figure 6:**
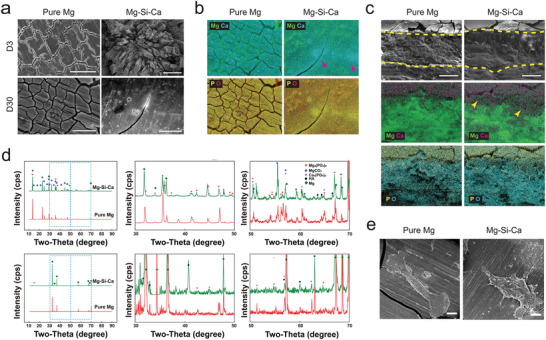
Formation of biomimicking bone–implant. a,b) Surface morphology a) and correspond EDX‐mapping b) of pure Mg and Mg–Si–Ca alloy after incubation in DMEM for different period of time under physiological condition (scale bar = 400 µm). c) Element distribution on the cross section of pure Mg and Mg–0.2Si–1.0Ca alloy after immersion in DMEM for 30 days (scale bar = 20 µm). d) XRD patterns of the pure Mg and Mg–0.2Si–1.0Ca alloy after immersed in DMEM for 7 days (upper) and 30 days (lower). e) Representative SEM images showing the attachment of hMSC on the surface of the implants 24 h after seeding (scale bar = 50 µm).

To investigate the difference in the degradation layer between the pure Mg and Mg–Si–Ca alloys at different time points, we further examined the degradation product of the implants under physiological condition in vitro using SEM and XRD. Mg–Si alloy was excluded from the following experiments due to its compromised in vivo performance. SEM images showed that the rod‐shaped crystals were first observed in the Mg–Si–Ca alloy at day 3 (**Figure** **6**a). The degradation layer on the Mg–Si–Ca alloy was thicker and more stable compared with that on pure Mg at day 30 (Figure 6a,c). Moreover, corresponding energy dispersive X‐ray spectroscopy (EDS) mapping showed that the degradation layers of Mg–Si–Ca alloy were richer in Ca and P than that of pure Mg, especially at the outer surface of the degradation layer (Figure 6b,c). Meanwhile, the presence of MgCO_3_, Mg_3_(PO_4_)_2_, and Ca_3_(PO_4_)_2_ in the degradation layers of Mg–Si–Ca alloy and pure Mg was verified by XRD, while hydroxyapatite (HA), a more stable phase, was only evident in the degradation product of Mg–Si–Ca alloy (Figure 6d). The cell attachment was evaluated by SEM (Figure 6e) and fluorescent staining (Figure S6c, Supporting Information) showed that hMSC appeared to be in a spindle shape with extension of pseudopodia when attached to the degradation layers of both pure Mg and Mg–Si–Ca alloy. This suggested that the degradation layers of both implants were favorable for cell attachment.

### 2.11 Osteogenic Property Induced by Integrin Signaling

**Figure 7:**
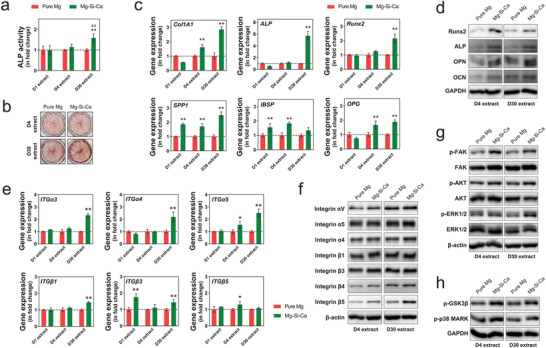
Osteogenic effect of Mg–Si–Ca implant through integrin signaling pathway. a,b) ALP activity a) (*n* = 3) and alizarin red staining b) (*n* = 3) of hMSC cultured in the medium extract of the pure Mg and Mg–Si–Ca alloy. c) The expression of bone marker genes of hMSC cultured in the medium extract of the pure Mg and Mg–Si–Ca alloy (*n* = 3)*, ** represents *p* < 0.05 and *p* < 0.01 compared with pure Mg group by one‐way ANOVA with Tukey's post hoc test. d) Representative western blots showing the expression of bone markers of hMSC cultured in the medium extract of the pure Mg and Mg–Si–Ca alloy. e) The gene expression of integrin subunits in hMSC cultured in the medium extract of the pure Mg and Mg–Si–Ca alloy. f) Representative western blots showing the effect of medium extract of the pure Mg and Mg–Si–Ca alloy on the expression of integrin subunits in hMSC. g) Representative western blots showing the phosphorylation of FAK, AKT, and ERK1/2 in hMSC in response to the stimulation of medium extract of the pure Mg and Mg–Si–Ca alloy. h) Representative western blots showing the phosphorylation of GSK3*β* and p‐38 MARK in hMSC in response to the stimulation of medium extract of the pure Mg and Mg–Si–Ca alloy.

We then conducted a series of in vitro tests to further evaluate the osteogenic potential of the microenvironment resulting from the degradation of the alloy. Since we have noticed that the ion release kinetics of the alloy could be significantly affected by the formation of degradation layers in vitro and in vivo, we propose that the ionic microenvironment of the implanted alloys and its influence on bone‐forming cells at different stages of the degradation can be distinct from each other. Therefore, we tested the osteogenic potential of the medium extract from the three different implants at day 1 (D1 extract), day 4 (D4 extract), and day 30 (D30 extract) of their degradation process. We found that, although there was no significant difference in the alkaline phosphatase (ALP) activity of hMSC cultured in D1 or D4 extract in the two implants, D30 extract of the Mg–Si–Ca alloy contributed to a 1.5‐fold increase in the ALP activity of hMSC compared with D30 extract of pure Mg (**Figure** **7**a). Although there was no significant difference in mineralization between hMSC cultured in D4 extract of pure Mg and Mg–Si–Ca alloy, the D30 extract of Mg–Si–Ca alloy was found to better induce mineralization of hMSC than that of pure Mg (Figure 7b).

Using real‐time quantitative polymerase chain reaction (RT‐qPCR) analysis, we found that D30 extract of Mg–Si–Ca alloy significantly upregulated the expressions of Collagen type I alpha 1 (*Col1A1*), ALP, runt‐related transcription factor 2 (*Runx2*), osteopontin (*OPN*), and osteoprotegerin (*OPG*) (Figure 7c). However, D1 extract and D4 extract of Mg–Si–Ca alloy contributed to significantly decreased expressions of *ALP* and *Col1A1*, as well as less than a twofold increase in the expressions of *OPN* and bone sialoprotein (*IBSP*). Interestingly, representative western blots showed that both D4 and D30 extracts of Mg–Si–Ca alloy promoted the expression of Runx2, ALP, OPN, and OCN in hMSC (Figure 7d), suggesting its superior osteogenic effects to the extract of pure Mg. As the activation of integrin subunits has been shown to play an important role in divalent cation‐induced differentiation of hMSC, we tested the expression of integrin subunits on the stimulation of medium extract from three alloys. Our RT‐qRCR data demonstrated that, compared with the D30 extract of pure Mg, the D30 extract of Mg–Si–Ca alloy significantly upregulated the expression of *ITGα3*, *ITGα4*, *ITGα5*, *ITGβ1*, and *ITGβ3* (Figure 7e). Instead, except for *ITGβ3*, there was no significant difference in the gene expression of integrin subunit when hMSC were treated with D1 extract of pure Mg or Mg–Si–Ca alloy. The D4 extract of Mg–Si–Ca alloy, however, only contributed to a marginal increase in the gene expression of *ITGα5* and *ITGβ5*. In addition, the effects of Mg–Si–Ca alloy were confirmed by western blotting because integrin *α*V, *α*4, *α*5, and *β*5 were found to be upregulated by the D30 extract of Mg–Si–Ca alloy but not the D4 one (Figure 7f). Meanwhile, the difference in the expression of integrin *β*3 was only seen when treated with D4 extract of pure Mg and Mg–Si–Ca alloy. We then investigated the downstream of integrin signaling and found that the extract of Mg–Si–Ca alloy, especially the D30 one, could contribute to significantly increased phosphorylation of focal adhesion kinase (FAK), protein kinase B (AKT), and extracellular‐signal‐regulated kinase (ERK) (Figure 7g). Consequently, the phosphorylation of glycogen synthase kinase‐3*β* (GSK3*β*) and p38 mitogen‐activated protein kinases (p38 MAPK) was also upregulated by the extract of Mg–Si–Ca alloy (Figure 7h).

### 2.12 Pro‐osteogenic Inflammatory Tissue Microenvironment

**Figure 8:**
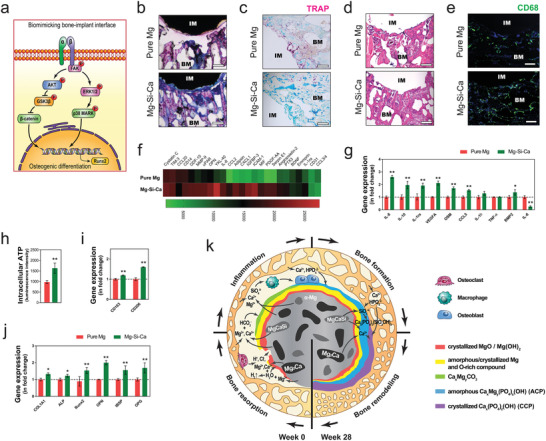
Pro‐osteogenic inflammatory tissue microenvironment that facilitates osteogenesis. a) The schematic diagram showing the activation of integrin signaling after the formation of biomimicking bone–implant interface. b–e) Representative Giemsa staining (b, scale bar = 500 µm), TRAP staining (c, scale bar = 200 µm), and H&E staining (d, scale bar = 200 µm) showing the early tissue response to the implants at week 1 postoperation. e) Representative immunofluorescent staining showing the presence of CD68 positive macrophage around the implants. f) Cytokine release profile of THP1‐derived macrophage cultured in the medium extract of the implants. g–i) The effect of medium extract on the inflammatory gene expression g), intracellular ATP level h), as well as gene expression of CD163 and CD206 i) in THP1‐derived macrophage. (*n* = 3), *p < 0.05 and **p < 0.01 by Student's *t*‐test . j) The effect of conditional medium from implant extract treated macrophage on the expression of osteoblast marker genes in hMSC (*n* = 3). k) The schematic diagram showing the dynamic degradation behaviors of the implants and their influences on bone healing process.

Besides the direct osteogenic effect of Mg–Si–Ca alloy on hMSC, we investigated the inflammatory response triggered by both implants because the difference in tissue response to the pure Mg and Mg–Si–Ca alloy can be seen as early as week one after the operation. Histological analysis using Giemsa staining revealed that the presence of these calcium‐based products in the degrading interface was closely related to tissue response to the implants (**Figure** **8**a). TRAP staining demonstrated that bone resorption around the Mg–Si–Ca implant was significantly attenuated when compared with the pure Mg implant (Figure 8c). H&E staining (Figure 8d) and immunofluorescent staining (Figure 8e) indicated that the acute inflammatory response triggered by the Mg–Si–Ca implant was characterized by a group of CD68 positive macrophages at the first week postoperatively. We then used cytokine array and RT‐qPCR assay to compare the level of inflammatory cytokines in macrophages upon the stimulation of pure Mg and Mg–Si–Ca extracts. The cytokines upregulated by the stimulation of Mg–Si–Ca extract included interleukin‐8 (IL‐8), C–C motif chemokine ligand 2 (CCL‐2), chemokine (C‐X‐C motif) ligand 1 (CXCL1), insulin‐like growth factor‐binding protein 3 (IGFBP‐3), intercellular adhesion molecule 1 (ICAM‐1), macrophage migration inhibitory factor (MIF), platelet‐derived growth factor (PDGF)‐AA, Serpin E1, Angiopoietin‐2, and Pentraxin‐related protein (PTX3) (Figure 8f). The inflammatory genes, including *IL‐8*, *IL‐10*, *IL‐1ra*, Vascular Endothelial Growth Factor (*VEGFA*), Oncostatin M (*OSM*), chemokine (C–C motif) ligand 5 (*CCL5*), and bone morphogenetic protein 2 (*BMP2*), were upregulated by the extract of the Mg–Si–Ca alloy. Moreover, *IL‐6* was significantly downregulated, while both *IL‐1β* and tumor‐necrosis factor alpha (*TNF‐α*) remained unchanged (Figure 8g). Our data also suggested that Mg–Si–Ca extract significantly increased the intracellular Adenosine triphosphate (ATP) activity of THP1‐derived macrophages (Figure 8h). Moreover, both CD163 and CD206, two markers for M2 polarization of macrophages, were found to be elevated in macrophages treated by the extract of Mg–Si–Ca alloy (Figure 8i). Using the conditional medium harvested from the implant extract stimulated macrophages, we further verified the osteogenic potential of the inflammatory microenvironment of the two implants to be different from each other. Indeed, the conditional medium from Mg–Si–Ca‐treated macrophages significantly promoted the osteogenic differentiation of hMSC, manifested by the upregulation of both early and late bone markers, including *COL1A1*, *ALP*, *OPN*, *IBSP*, *OPG*, and *Runx2* (Figure 8j).

## 3 Discussion

### 3.1 Dynamic Biodegradation Process

The intrinsic difference between Mg–Si–Ca alloy and the other two implants is the presence of the second phase. In this study, Mg_2_Ca in Mg–Si–Ca alloy is recognized as an anode in the Mg–Mg2Ca galvanic couple,^[^ ^27^, ^28^ ^]^ while MgCaSi acts as a cathode in the Mg–MgCaSi couple.^[^ ^29^ ^]^ Therefore, the Mg_2_Ca phase is supposed to degrade before the Mg matrix, followed by the degradation of the MgCaSiphase (Equations (1)–(4))^[^ ^30^ ^]^

(1) $$Mg_{2}\mathrm{Ca}\to 2Mg^{2+}+Ca^{2+}+6e^{-\;}$$

(2) $$\mathrm{MgCaSi}+4H_{2}O\;\to Mg^{2+}+Ca^{2+}+\mathrm{Si}O_{2}+4H^{+}+8e^{-}$$

(3) $$Ca^{2+}+\;OH^{-}\;↔\;\mathrm{Ca}{\left(\mathrm{OH}\right)}_{2}$$

(4) $$\mathrm{Si}O_{2}+4OH^{-}\;\to {\mathrm{SiO}}_{4}^{4-}+2H_{2}O$$

Generally, galvanic corrosion resulting from the second phase is supposed to accelerate the degradation of the Mg alloy. Although we observed rapid degradation of Mg–Si–Ca alloy as compared with the results of pure Mg in the in vitro immersion test (Figure 1e,f), our in vivo degradation result obtained by micro‐CT scan and histology analysis (Figures 1f and 2c) suggested that the degradation rate of Mg–Si–Ca alloy was significantly slower than that of the other two implants, in particular at the late stage. The discrepancy between Mg–Si–Ca alloy and the other two implants in terms of material degradation measured by in vitro and in vivo experiments may be attributed to the variations between the static cell culturing environment and the dynamic in vivo tissue environment.^[^ ^17^ ^]^ Indeed, when the immersing medium was continuously refreshed over a period of 1 month, a gradual change in the degradation rate of Mg–Si–Ca alloy could be noted at around day 21 (Figure 1g). These data, together with the SEM images (Figure 6c) and XRD patterns (Figure 6d), showing the dynamic changes in the surface degradation products of Mg–Si–Ca alloy, implies that the degradation rate of the implant is not only determined by its intrinsic properties but also influenced by the degradation layer formed through the interaction with the adjacent tissue microenvironment.

To further understand the dynamic degradation behavior of the implants in relation to the formation of degradation layers in vivo, we adopted multiple methods to characterize the degradation products of pure Mg, Mg–Si alloy, and Mg–Si–Ca alloy over the 28 weeks after their implantation in rat femurs. The degradation of Mg‐based materials starts with their direct contact with aqueous solution, through which MgO and Mg(OH)_2_ compounds form on the surface as the result of the anodic reactions, cathodic reaction, and precipitation reaction. This Mg‐ and O‐rich layer was found on all sample surfaces (Figure 4b; and Figure S6a, Supporting Information). The crystalline MgO and Mg(OH)_2_ compounds in the thickness of hundreds of nanometers were found on the surface of the implants during the whole implantation period (Figure 5c–h). A relatively thicker layer in its several microns composed of amorphous Mg‐ and O‐rich compounds MgO and Mg(OH)_2_ was observed underneath the crystalline layer as reported elsewhere.^[^ ^13^ ^]^ However, the layers of MgO and Mg(OH)_2_ compounds were unable to protect the degradable implants against the corrosion initiated by surrounding body fluid, which was evidenced by the gas production and significant bone resorption near the implant at the early stage of implantation (Figure 2a,b,f). Due to the fast degradation of implants and the rapid change in the peri‐implant tissue microenvironment, osteoclasts were activated to dissolve bone minerals, followed by the degradation of bone matrix proteins, leading to the accumulation of H^+^, Cl^−^, and HCO_3_^−^ in the peri‐implant area.^[^ ^31^ ^]^ Moreover, both MgO and Mg(OH)_2_ became more vulnerable when acidic bone resorption was initiated by osteoclasts. Therefore, the protection of the material matrix from corrosive body fluid would only be achieved after a more durable bone–implant interface was formed.

Following the establishment of the initial degradation layers consisting of MgO and Mg(OH)_2_, the bone–implant interface was gradually matured with the liberated Ca^2+^, HCO_3_^−^ and HPO_4_^2−^ from bone marrow and circulation through the series of chemical reactions listed below. In brief, HCO^3−^ and HPO_4_^2−^ can convert the Mg(OH)_2_ (Ksp = 5.6 × 10^−12^) into MgCO_3_ (Ksp = 6.8 × 10^−6^) and Mg_3_(PO_4_)_2_ (Ksp = 1.04 × 10^−24^), which could be detected on the surface of pure Mg and Mg–Si–Ca alloys after 7 and 30 days of immersion in DMEM (Figure 3). Then, the free Ca^2+^ present in the peri‐implant microenvironment can further react with OH^−^, HCO_3_^−^, and HPO_4_^2−^ to form Ca(OH)_2_ (Ksp = 4.7 × 10^−6^), CaCO_3_ (Ksp = 5.0 × 10^−9^), Ca_3_(PO_4_)_2_ (Ksp = 2.1 × 10^−33^), and Ca_5_(PO_4_)_3_(OH) (Ksp = 2.2 × 10^−61^). ^[^ ^32^ ^]^ This process is more prominent in Mg–Si–Ca implant because the degradation of its second phases (i.e., Mg_2_Ca and MgCaSi) contributed to early enrichment of Ca^2+^ in the bone–implant interface (Figures 4 and 5)

(8) $$\begin{array}{c} \mathrm{HC}{O_{3}}^{-}+\mathrm{Mg}{\left(\mathrm{OH}\right)}_{2}/\mathrm{Ca}{\left(\mathrm{OH}\right)}_{2}↔\mathrm{MgC}O_{3}/\mathrm{CaC}O_{3}+H_{2}O+OH^{-} \end{array}$$

(9) $$\begin{array}{ccc} & & 2\mathrm{HP}{O_{4}}^{2-}+3\mathrm{Mg}{\left(\mathrm{OH}\right)}_{2}/\mathrm{Ca}{\left(\mathrm{OH}\right)}_{2}↔Mg_{3}{\left(PO_{4}\right)}_{2}/Ca_{3}{\left(PO_{4}\right)}_{2}\\ & & \;+2H_{2}O+4OH^{-} \end{array}$$

(10) $$2\mathrm{HP}{O_{4}}^{2-}+\;3Ca^{2+}+\;2OH^{-}\;↔Ca_{3}{\left(PO_{4}\right)}_{2}+2H_{2}O$$

(11) $$3\mathrm{HP}{O_{4}}^{2-}+5Ca^{2+}+4OH^{-}\;↔Ca_{5}{\left(PO_{4}\right)}_{3}\left(\mathrm{OH}\right)+3H_{2}O$$

(12) $$\begin{array}{ccc} & & 3\mathrm{HP}{O_{4}}^{2-}+\;9Ca^{2+}+3{\mathrm{SiO}}_{4}^{4-}+\;6OH^{-}\;↔Ca_{9}{\left(PO_{4}\right)}_{3}{\left(\mathrm{Si}O_{3}\mathrm{OH}\right)}_{3}\\ & & \;+3H_{2}O \end{array}$$

### 3.2 The Assembling of Biomimicking Bone–Implant Interface

The composition and microstructure of the bone‐to‐implant interface determine the host response, which is thought to be the key factor in the clinical success of biodegradable Mg implant.^[^ ^9^, ^13^ ^]^ The degradation products, which are found to mainly contain oxygen, magnesium, calcium, and phosphorous,^[^ ^7^, ^33^ ^]^ could be resorbed by osteoclasts and then participate in new bone formation mediated by osteoblasts.^[^ ^13^ ^]^ More importantly, newly formed bone tissue could integrate with Mg alloys through the bone‐to‐implant interface due to the similar composition between degradation layers and mineral bone.^[^ ^18^ ^]^ Indeed, the formation of a biomimicking calcification matrix at the degrading interface is essential for regulating the degradation rate of the Mg alloy and initiating the substitution of the degrading implant by newly formed bone tissue. In this study, our results suggested that the simultaneous incorporation of Si and Ca in Mg–Si–Ca alloy accelerated the formation of the biomimicking calcification matrix that contributed to the early onset of the pro‐osteogenic tissue microenvironment.

Using of the TEM technique, we further analyzed the nanostructure of the bone–implant interface and identified two distinct regions in the Ca–P layer based on the degree of crystallinity. The amorphous calcium phosphate (ACP) layer near the amorphous Mg(OH)_2_ layer was evident in both pure Mg and Mg–Si–Ca alloy. However, the content of Ca and P within this layer in the pure Mg sample was comparatively lower than that of the Mg–Si–Ca alloy after 28 weeks of implantation (Figure 5). The presence of osteoconductive ACP in the bone–implant interface is essential for the osseointegration process because it can serve as a precursor for biomineralization that facilitates the assembling of hydroxyapatite nanoparticles into highly ordered structures.^[^ ^34^ ^]^ Moreover, in the Mg–Si–Ca group, but not the pure Mg group, a gradual change of the degradation product from amorphous ACP to highly ordered crystalline calcium phosphate compounds was observed on the outer layer of the interface. This can be attributed to the presence of SiO_4_^4−^ in the peri‐implant microenvironment after the degradation of MgSiCa phase, because a trace amount of Si can promote the crystallization of calcium phosphate.^[^ ^22^ ^]^ The results of XRD and TEM analysis further confirmed that the crystalline calcium phosphate is hydroxyapatite (HA), which is more stable and resistant to the attack of body fluid.^[^ ^8^, ^35^ ^]^ Hence, the degradation rate of Mg–Si–Ca alloy was significantly reduced after the interface was stabilized. The formation of bone‐like apatite also contributes to osseointegration due to the structural similarity between the crystalline calcium phosphate layer and the bone minerals. In contrast, the ACP layer found in pure Mg failed to develop into crystalline calcium phosphate without the participation of Si, leaving the implant and its layers vulnerable to continuous corrosion initiated by corrosive body fluid and osteoclastic activity.

### 3.3 The Pro‐Osteogenic Tissue Microenvironment

Integrins consist of two transmembrane glycoprotein subunits, the *α* chain and the *β* chain, which are noncovalently bound. They are extensively studied in the biomaterial field as linker proteins between osteoblasts and orthopedic biomaterials.^[^ ^36^ ^]^ The integrin subunits regulate cellular behavior through bidirectional signaling pathways: they sense and respond to the external stimuli with their extracellular domains, whereas the intracellular domains anchor to cytoskeletal proteins and link with signaling molecules to regulate various cellular functions such as cell adhesion, proliferation, and differentiation.^[^ ^36^ ^]^ In this study, we demonstrated that the expressions of several integrin subunits were altered by the degradation extracts of Mg–Si–Ca implant. For instance, integrin *α*3 and *α*V as indicators for osteoblast differentiation were both upregulated by the extracts of Mg–Si–Ca alloy.^[^ ^37^ ^]^ Meanwhile, the same extracts also promoted the expression of integrin *α*4, which is uniquely expressed in osteoblasts.^[^ ^38^ ^]^ In addition, the expressions of integrin *α*5 and *β*5 that contribute to the osteoblast differentiation and osteogenic capacity of hMSC were also elevated by the medium extract of Mg–Si–Ca alloy.^[^ ^39^, ^40^ ^]^

The first step of integrin signaling activation is the autophosphorylation of focal adhesion kinase (FAK) in, which contributes to further tyrosine phosphorylation and recruitment of other proteins.^[^ ^41^, ^42^ ^]^ Indeed, FAK plays a crucial role in integrating integrin activation into a variety of signaling cascades contributing to osteogenic differentiation of osteoblasts,^[^ ^43^ ^]^ whereas the loss of FAK function results in delayed bone healing.^[^ ^44^ ^]^ As compared with pure Mg, we showed that the Mg–Si–Ca sample significantly enhanced the phosphorylation of FAK, as well as AKT and ERK1/2, which are two major downstream of FAK signaling involved in osteogenesis. Moreover, we observed that Mg–Si–Ca extracts could promote the phosphorylation of their downstream proteins, GSK3*β*, and p38 MAPK. The phosphorylation of AKT followed by the phosphorylation GSK3*β* indicates the activation of the WNT signaling pathway, leading to the nuclear translocation of *β*‐catenin and the expression of target genes by binding to Lef1/Tcf transcription factors.^[^ ^45^ ^]^ This observation is consistent with our finding in the upregulation of integrin *α*5 by Mg–Si–Ca, as the AKT‐GSK3*β* signaling‐dependent osteogenic differentiation of hMSC is mediated by integrin *α*5.^[^ ^40^ ^]^ In addition, the activation of ERK1/2‐p38 MAPKs signaling pathway mediated by integrin *α*5 can contribute to the osteogenic differentiation of hMSC by targeting the Runx2 gene.^[^ ^46^, ^47^ ^]^

In addition to the direct effect of Mg–Si–Ca extract on hMSC through integrin signaling, the altered peri‐implant tissue microenvironment resulting from the formation of degradation layers also contributed to osteogenesis via regulating the macrophage‐mediated inflammation response. It has been reported that the rapid corrosion of Mg‐based materials could induce excessive inflammatory response leading to fibrosis formation that delayed the bone healing process.^[^ ^48^ ^]^ Therefore, various surface coatings designed to suppress Mg corrosion may attenuate proinflammatory gene expressions.^[^ ^48^ ^]^ In this study, we demonstrated that the self‐assembled bone–implant interface contributed to a peri‐implant microenvironment that would not significantly induce the proinflammatory cytokines of macrophages, such as IL‐1*β*, TNF‐*α*, and IL‐6. In contrast, a series of pro‐osteogenic cytokines that could trigger the osteogenic potentials of hMSC (e.g., CCL5 and IL‐8),^[^ ^49^, ^50^ ^]^ were elevated by the Mg–Si–Ca extract. Meanwhile, the proangiogenic cytokines VEGFA and PDGF‐AA and the anti‐inflammatory cytokines IL‐10 and IL‐1ra are upregulated by Mg–Si–Ca extract, indicating its effects on angiogenesis and osteogenesis.^[^ ^51^, ^52^, ^53^, ^54^ ^]^ Therefore, we believe that the tissue microenvironment created during the degradation of Mg–Si–Ca alloy favorably triggers in situ new bone formation.

## 4 Conclusion

In summary, we demonstrated that the addition of Ca and Si to Mg accelerated the formation of a self‐assembled, multilayered implant–tissue interface, which coordinates the biodegradation of the implant with the bone healing process. At the initial stage of implantation, a burst release of Mg^2+^ from Mg–Si–Ca alloy activated the monocyte–macrophage lineage, leading to an immune microenvironment favorable for the recruitment of MSCs and initiation of osteogenic differentiation. With the formation of the biomimicking calcified matrix at the degrading bone–implant interface, the ion release kinetics of the Mg–Si–Ca alloy was turned down, leading to a new peri‐implant microenvironment for osseointegration and osteogenesis by targeting the integrin signaling pathways in MSCs. Our study revealed that the spontaneously formed degradation layers at the bone–implant interface are the key to the superior performance of Mg–Si–Ca alloy, as they regulate the biodegradation behaviors of the implant to elicit an appropriate host response at different stages of bone healing.

## 5 Experimental Section

### Experimental Design

The aim of this study was to design a Mg‐based alloy that allows early osteointegration and gradual substitution by host bony tissue. A series of Mg–*x*Si and Mg–*x*Si–*y*Ca alloys were developed and their microstructures, degradation behaviors, mechanical properties, and biocompatibility in vitro were tested. Then, Mg–0.2Si and Mg–0.2Si–1.0Ca were selected and its osteogenic properties in a rat model compared with pure Mg were evaluated. Moreover, several methods were adopted, including SEM, TEM, and XRD, to study the in vitro and in vivo degradation behavior of the alloy. The composition of the bone–implant interface at the nanoscale after the implantation of the alloy in rat femurs to confirm its bioadaption to bony tissue was analyzed.

### Alloy Preparation

A vacuum induction furnace (ZG‐0.01) was used to melt the alloys under a protective atmosphere of Ar. Raw materials consisted of high pure magnesium (99.99%), silicon (99.99%), and calcium (99.95%). A tantalum crucible and high purity graphite mold were used during the material preparation to keep the impurities as low as possible. The raw materials were molten at 750 °C and maintained for 15–20 min to allow the uniform dispersion of the alloying elements before casting. The compositions and impurities of the materials after solidification (listed in **Table** **1**) were measured by inductively coupled plasma atomic emission spectroscopy (ICP‐AES, JY38S). All the materials were then extruded at 320 °C with an extrusion rate of 2 mm s^−1^ and an extrusion ratio of 1/16.

**Table 1:** Chemical compositions of the experimental materials measured by ICP‐AES

Alloy	Composition [wt%]						
	Si	Ca	Be	Fe	Ni	Cu	Mg
Mg–0.2Si–1.0Ca	0.17	0.97	0.00 019	0.0063	0.00 048	0.00 086	Balance
Mg–0.2Si–0.5Ca	0.18	0.47	0.00 022	0.0067	0.00 041	0.00 075	Balance
Mg–0.4Si–1.0Ca	0.37	1.05	0.00 018	0.0062	0.00 049	0.00 073	Balance
Mg–0.4Si–0.5Ca	0.38	0.49	0.00 024	0.0056	0.00 053	0.00 068	Balance
Mg–1.0Si–1.0Ca	0.95	1.12	0.00 020	0.0067	0.00 058	0.00 071	Balance
Mg–1.0Si–0.5Ca	0.98	0.52	0.00 015	0.0060	0.00 051	0.00 066	Balance

### Microstructure Characterization

Disk‐shaped specimens with a thickness of 2 mm and a diameter of 10 mm were cut from the as‐extruded rods. These specimens were ground up to 5000 grit and polished in a solution containing 20 mL of glycerol, 2 mL of hydrochloric acid, 3 mL of nitric acid, and 5 mL of acetic acid. The microstructure was observed using an environmental scanning electron microscope (ESEM, FEI Quanta 200F, USA) equipped with an energy dispersive X‐ray spectroscopy (EDS) detector at an accelerating voltage of 15 kV. In addition, the second phases were detected by XRD (Rigaku DMAX 2400, Japan) using Cu K*α* radiation (40 kV, 100 mA) at a step size of 0.02° from 10° to 90°. Phase identification was performed using JADE software (MDI Inc., Livermore, CA).

### Degradation Behavior Test In Vitro

Specimens with a diameter of 10 mm and a thickness of 1.5 mm were ground up to 2000 grit before the immersion test. They were immersed in Hank's solution with an exposure ratio of 20 mL cm^−2^ according to ASTM‐G31‐72. The pH value was tested by a pH meter at the designated time points. At each designated time point, at least three samples were removed from the solution and washed by a chromic acid cleaning solution containing 200 g L^−1^ CrO_3_ and 2 g L^−1^ AgNO_3_ to remove the degradation products. Subsequently, the specimens were washed with deionized (DI) water, air dried, and weighed. The degradation rate was calculated based on the following equation

(13) $$\mathrm{weight}\;\mathrm{loss}\;\mathrm{ratio}\;=\frac{w_{0}-w_{1}}{w_{0}}\;\times \;100\%$$

*w*_0_: initial weight *w*_1_: weight at the designated time point

The three‐electrode cell configuration with a counter electrode (CE) made by a platinum foil and a saturated calomel electrode (SCE) was used for the electrochemical test. Experimental samples with an exposed area of 0.45 cm^2^ acted as the working electrode. Hank's solution was used as the electrolyte. Measurements were carried out on an electrochemical workstation (PGSTAT 302N, Metrohm Autolab). The open circuit potential (OCP) was continuously monitored for 3600 s. Potentiodynamic polarization tests were performed at a scanning rate of 1 mV s^−1^. Electrochemical parameters and corrosion rates were calculated according to the ASTM‐G102‐89 standard. At least three duplicates of each material were tested for statistical analysis.

### Cytotoxicity Assay

Cytotoxicity evaluation of the materials was conducted by indirect cell culture using mouse preosteoblasts MC3T3‐E1 and human TERT‐immortalized mesenchymal stem cell (hMSC). The extract was prepared by immersing the sample in DMEM (ThermoFisher Scientific) supplemented with 10% fetal bovine serum and 1% penicillin/streptomycin (P/S) with an extraction ratio of 1 cm^2^ mL^−1^ under standard cell culture conditions (i.e., 37 °C, 5%/95% CO_2_/air, humidified sterile environment). Hereafter, DMEM + 10% FBS +1% P/S is referred to as DMEM. After 24 h, the supernatant solution was harvested by centrifugation and subjected to cell culture. MC3T3‐E1 cells or hMSC were seeded on a 96‐well tissue culture plate at a density of 3 × 10^3^ cells per well. After overnight attachment, the culture medium was replaced by 25% or 50% diluted extract. Cells cultured with normal cultural medium served as a negative control. After incubation for 1, 3, and 5 days, the medium was replaced by DMEM containing a 10% mitochondrial activity‐based cell counting kit (CCK‐8, Dojindo) after washing with phosphate‐buffered saline (PBS) three times. DMEM containing 10% CCK‐8 without cells was used as a blank control. After 1 h of incubation, the absorbance (optical density, OD) was measured using the multimode detector (BioRad 680) at a wavelength of 450 nm. The cell viability was calculated as follows:

(14) $$\mathrm{cell}\;\mathrm{viability}=\frac{\mathrm{OD}\;\left(\mathrm{testing}\;\mathrm{group}\right)-\mathrm{OD}\;\left(\mathrm{blank}\;\mathrm{control}\right)}{\mathrm{OD}\;\left(\mathrm{negative}\;\mathrm{control}\right)-\mathrm{OD}\;\left(\mathrm{blank}\;\mathrm{control}\right)}\times 100\%$$

### Mechanical Tests

Following ASTM E8‐04, tensile specimens with a gauge length of 25 mm, width of 6 mm, and thickness of 2 mm were machined parallel to the extrusion direction. Tensile tests were carried out at a crosshead speed of 1 mm min^−1^ and room temperature using a universal material testing machine (Instron 5969) and an extensometer with a gauge length of 25 mm. At least three samples of each material were tested.

### Ion Release Profile In Vitro

Pure Mg, Mg–0.2Si alloy, and Mg–0.2Si–1.0Ca alloy were immersed with an exposure ratio of 1 cm^2^ mL^−1^ in DMEM at 37 °C in a humidified atmosphere with 5% CO_2_. At designated time points, the culture medium was replenished with fresh DMEM, and the concentrations of Mg^2+^, Ca^2+^, and SiO_4_^4−^ ions in the medium were measured using inductively coupled plasma optical emission spectrometry (ICP‐OES, Perkin Elmer, Optima 2100DV). Therefore, the ion release data shown represent the accumulated ion released from the alloy between two designated time points for medium refreshment.

### Degradation Layer Analysis In Vitro

The surface morphology of pure Mg and Mg–0.2Si–1.0Ca alloy after immersion in DMEM was observed using SEM (S‐4800, Hitachi). The distributions of Mg, Ca, O, and P on the surface and cross‐section of the degradation layer were analyzed using SEM‐EDX mapping. Moreover, the degradation products on the sample were examined using XRD and analyzed by JADE software.

### Cell Attachment Assay

The cell attachment of hMSC on alloys was evaluated by SEM (S‐4800, Hitachi) and immunocytochemistry analysis. For SEM analysis, hMSC were seeded directly on the DMEM preimmersed disk‐shaped alloy and left overnight to allow attachment. The attached cells were fixed with 2.5% glutaraldehyde at 4 °C overnight and dehydrated with gradient alcohols. Finally, the samples were dried using a critical point dryer (HCP‐2, Hitachi) and coated with a gold sputter (E1010, Hitachi ion sputter) before SEM observation. For immunocytochemistry analysis, attached cells were washed with PBS, fixed with 4% paraformaldehyde, and permeabilized with 0.2% Triton X‐100. The cytoskeletons were stained with FITC‐Phallotoxins (Sigma‐Aldrich), while the nuclei were stained with Hoechst 33 342 (ThermoFisher Scientific).

### Animal Surgery

Pure Mg, Mg–0.2Si, and Mg–0.2Si–1.0Ca alloys were prepared as cylindrical rods with a diameter of 2.2 mm and a length of 6.0 mm. The rods were further polished to a diameter of 2.0 mm and sterilized under Co60 *γ* ray radiation at 25 KGy before implantation. The animal experiments were authorized by the Licensing Office of the Department of Health of the Hong Kong Government and University Ethics Committee of the University of Hong Kong. All surgical procedures, including anesthetic, operation, and postoperative care, were approved by the University Ethics Committee of the University of Hong Kong. Twelve‐week‐old female Sprague‐Dawley (SD) rats were purchased from the Laboratory Animal Unit of the University of Hong Kong and maintained in a specific pathogen‐free condition. Forty‐six rats were divided into two groups of 23 rats each, and 6 other rats were used as a sham group. Prior to operation, rats were anesthetized with a combination of xylazine (6 mg kg^−1^) and ketamine (67 mg kg^−1^) through intraperitoneal injection. Buprenorphine (0.05 mg kg^−1^) was administered subcutaneously to minimize the suffering of animals. A tunnel defect of 2 mm in diameter was prepared at the femur lateral epicondyle using a hand driller. Subsequently, alloy rods made of pure Mg and Mg–0.2Si–1.0Ca alloy were implanted into the prepared tunnel defects. In the sham group, no implant was placed in the defect. The wound was sutured layer‐by‐layer. Oxytetracycline (60 mg kg^−1^) was administered subcutaneously as an antibiotic prophylactic every 72 h. Flunixin (2.5 mg kg^−1^) was subcutaneously injected every 12 h for 3 days after surgery.

### Fluorochrome Labeling

For evaluating the dynamic bone formation process, two fluorochrome labels were used to label the newly formed bone tissues. In brief, calcein green (5 mg kg^−1^, Sigma‐Aldrich) was subcutaneously injected into rats 2 weeks after the surgery. xylenol orange (90 mg kg^−1^, Sigma‐Aldrich) was injected 3 weeks after the surgery. The fluorochrome labels were captured using a fluorescence microscope (Nikon, Tokyo, Japan) from the nondecalcified sections that were detailed in the following parts.

### Radiographic Evaluation

X‐ray radiography (Faxitron X‐ray Corporation, USA) was used to monitor the surgical outcome immediately after the surgery and the healing process at each time point (i.e., weeks 2, 4, 8, 12, 15, 24, and 28) after the operation. In addition, the degradation of alloy and the new bone formation were evaluated using a live animal micro‐CT scanning device (SkyScan 1076, Kontich, Belgium) at these designated time points. A resolution of 17.33 µm, a voltage of 88 kV, and an amperage of 100 µA were selected to obtain the CT image. Two phantoms containing rods with standard densities of 0.25 and 0.75 g cm^−3^ were scanned with each sample for calibration. The CT data were reconstructed and analyzed using CTAn (Skyscan Company), and the 3D models were generated by CTVol (Skyscan Company).

### Histological Analysis

At weeks 1, 2, 4, 8, and 28 after the operation, rats were euthanized for the preparation of tissue sections. For nondecalcified sections, the femur implanted with the alloy was fixed in 10% buffered formalin for 48 h and then dehydrated with gradient ethanol. Xylene was used as a transition before embedding the specimens in methyl‐methacrylate (MMA). Then, the embedded samples were cut into slices with a thickness of ≈200 µm and ground to a thickness of 40–60 µm. The selected sections were stained with either Giemsa (Merck, Germany) staining or Goldner's trichrome (Sigma‐Aldrich, USA) staining. For decalcified sections, the femur was decalcified in 10% ethylenediaminetetraacetic acid (EDTA, Sigma‐Aldrich, USA) solution for 6 weeks. The implant was carefully explanted from the femur. The femur was then dehydrated in gradient ethanol and embedded in paraffin. The slices were obtained by cutting the samples into slices with a thickness of 5 µm using a rotary microtome (RM215, Leica Microsystems, Germany). H&E staining and TRAP staining (Sigma‐Aldrich, USA) were performed on selected slides from each sample following the manufacturer's instructions. Images of the stained sections were captured using a polarizing microscope (Nikon, Tokyo, Japan). Immunostaining was performed using Proteinase K (Sigma‐Aldrich) for proteolytic digestion and 3% H_2_O_2_ for the elimination of endogenous peroxidase activity. The primary antibodies used in this study include rabbit anti‐CD68 (Abcam, USA) and rabbit anti‐OCN (Abcam, USA). The immunofluorescent images were captured using confocal microscopy (LSM 780, Zeiss, Germany).

### Characterization of the Implant–Tissue Interface

The surface topography and elemental composition of implants retrieved from the rats euthanized at 12 weeks postoperatively were characterized using ESEM and EDS, respectively. Elemental distribution maps on the surface of dissected implants were obtained. XRD was used to analyze the degradation products or mineral depositions on the implant surface. The undecalcified sections were used to analyze the degradation layer between the implant and bone. The micromorphology and elemental distribution at the bone–implant interface were observed using ESEM and EDX at an accelerating voltage of 10 kV. The TEM (Tecnai G2 F20) was used to analyze the composition and crystallinity of the layers between implant and bone. The samples were prepared using focus ion beam (FIB, FEI Strata DB 235). Then bright‐field images and SAED patterns were captured by TEM at a voltage of 200 kV. Besides, Fourier transform infrared spectroscopy reflection (μFTIR) was performed with a Spectrum Spotlight 200 FT‐IR microscopy (PE) on the transition area between bone and implant, and the spectra were recorded from 4000 to 560 cm^−1^ in a square area with a width of 150 µm. OMNIC software was utilized to fit and calculate the characteristic peak of functional groups.

### ALP and Mineralization Assay

The osteogenic effect of the extract was determined by the ALP activity of hMSC using the para‐Nitrophenylphosphate (pNPP) method. At the designated time points, the cells were lysed with 0.2% Triton X‐100 at 4 °C for 30 min. The supernatant of the lysis was collected by centrifugation and assayed using an ALP detection kit (Sigma‐Aldrich, USA) following the manufacturer's instructions. The total protein content in the supernatant was measured using a bicinchoninic acid (BCA) Protein Assay Kit (ThermoFisher Scientific). The relative ALP activity was normalized to total protein content and expressed as units per gram of protein. The calcium nodules were stained using Alizarin red staining to study the mineralization of the extracellular matrix. On day 21 after osteogenic induction, cells were fixed with 4% paraformaldehyde for 15 min and thoroughly washed three times with PBS. The mineralization nodules were stained with 1% Alizarin Red solution at pH 4.1. After thorough washing with Millipore water, the sample was air dried in air before it was photographed.

### RT‐qPCR Assay

For real time quantitative PCR (RT‐qPCR) assay, the total RNA of the cells was extracted and purified using an RNeasy Plus kit (Qiagen) following the manufacturer's instructions. For the reverse transcript, complementary DNA was synthesized using Takara RT Master Mix (Takara) following the manufacturer's instructions. The primers used for the RT‐qPCR assay were synthesized by Life Technologies (ThermoFisher Scientific) based on sequences retrieved from Primer Bank (http://pga.mgh.harvard.edu/primerbank/). SYBR Green Premix Ex Taq (Takara) was used for the amplification and detection of cDNA targets on a StepOne Plus Realtime PCR system (Applied Biosystems). The mean cycle threshold (Ct) value of each target gene was normalized to the housekeeping gene glyceraldehyde 3‐phosphate dehydrogenase (GAPDH). The results were shown in a fold change using the ∆∆Ct method.

### Western Blotting

For western blotting assay, total protein was lysed with RIPA Lysis and Extraction Buffer (ThermoFisher Scientific) after three washes with ice‐cold PBS. The supernatants were collected by centrifugation at 15 000 × g for 10 min at 4 °C. The protein concentration was measured with a BCA Protein Assay Kit (ThermoFisher Scientific). A total of 30 µg of protein from each sample was subjected to SDS‐PAGE electrophoresis and transferred to PVDF membrane (Merck Millipore). Then, the membrane was blocked in 5% w/v bovine serum albumin (BSA, Sigma‐Aldrich) and incubated with blocking buffer diluted primary antibodies overnight at 4 °C. The primary antibodies used, including rabbit anti‐integrin *α*5, rabbit anti‐integrin *α*4, rabbit anti‐integrin *α*V, rabbit anti‐integrin *β*1, rabbit anti‐integrin *β*3, rabbit anti‐integrin *β*4, rabbit anti‐integrin *β*5, rabbit anti‐p‐FAK, rabbit anti‐FAK, rabbit anti‐p‐AKT, rabbit anti‐AKT, rabbit anti‐p‐ERK1/2, rabbit anti‐ERK1/2, rabbit anti‐p‐GSK3*β*, rabbit anti‐p‐p38 MAPK, rabbit anti‐*β*‐actin, and rabbit anti‐GAPDH, were purchased from Cell Signaling Technology. The other antibodies, including rabbit anti‐Runx2, anti‐ALP, anti‐OPN, and anti‐OCN, were purchased from Abcam (USA). After incubation with goat anti‐rabbit immunoglobulin G (IgG) antibody for 1 h, the protein bands were visualized by electrochemiluminescence substrate (ThermoFisher Scientific) and exposed under the ChemiDoc XRS System (BioRad).

### Nanoindentation

The nanoindentation experiment was carried out with a Tribo Indenter (Hysitron, USA) on poly(methyl methacrylate)‐embedded bone slices. The Berkovich diamond pyramid tip was operated in load‐controlled static mode with a loading rate of 40 µN s^−1^ to a maximum loading of 400 µN. The indenter was held at maximum load for 2 s before unloading at a rate of 40 µN s^−1^. The Oliver–Pharr method was used to determine the modulus and hardness.^[^ ^55^ ^]^ Three to five indents were performed at each position, which is at a specific distance (i.e., 50, 100, 150, 250, 350, 450, 650, and 850 µm) from the implant.

### Statistical Analysis

All the experiments in this work were repeated at least three times, and the results were expressed as means ± standard deviations. Differences between groups were analyzed using one‐way analysis of variance (ANOVA) followed by the Tukey test. The levels of significant differences were presented as * *p* < 0.05 and ** *p* < 0.01.

## Conflict of Interest

The authors declare no conflict of interest.

## Authors Contribution

W.L. and W.Q. contributed equally to this work. W.L. and W.Q. conducted the animal surgery, histology study, as well as the in vitro and in vivo tests. X.L. and D.B. helped with the preparation of Mg alloys. D.S., T.M.W., and K.K. helped with the data analysis. K.W.K.Y., Y.Z., and K.M.C.C. contributed to data interpretation and supervised the project. W.Q. and W.L. wrote the manuscript with input from all authors.

## Supporting information

Supporting Information

## Data Availability

Data available on request from the authors.
