# Supplementary material for: Biomimicking Bone–Implant Interface Facilitates the Bioadaption of a New Degradable Magnesium Alloy to the Bone Tissue Microenvironment
Source: Adv Sci (Weinh). 2021 Oct 28;8(23):2102035. doi: 10.1002/advs.202102035 (PMC8655172; doi:10.1002/advs.202102035)
Supplement: Supplementary file 1 — Supporting Information [file ADVS-8-2102035-s001.pdf]

WILEY-VCH

© 2021 Wiley-VCH GmbH

ADVANCED SCIENCE
Open Access

Supporting Information

for *Adv. Sci.*, DOI: 10.1002/advs.202102035

Biomimicking Bone–implant Interface Facilitates the Bio-adaption of a New Degradable Magnesium Alloy to the Bone Tissue Microenvironment

Wenting Li[1,2,4†], Wei Qiao[2,4†], Xiao Liu[1], Dong Bian[3], Danni Shen[1,4], Yufeng Zheng[1], Jun Wu[4], Kenny Y. H. Kwan[2,4], Tak Man Wong[2,4], Kenneth M. C. Cheung[2,4], Kelvin W. K. Yeung[2,4*]*

Supporting Information

Biomimicking bone–implant interface facilitates the bio-adaption of a new degradable magnesium alloy to the bone tissue microenvironment

Wenting Li[1,2,4†], Wei Qiao[2,4†], Xiao Liu[1], Dong Bian[3], Danni Shen[1,4], Yufeng Zheng[1], Jun Wu[4], Kenny Y. H. Kwan[2,4], Tak Man Wong[2,4], Kenneth M. C. Cheung[2,4], Kelvin W. K. Yeung[2,4*]*

((Please insert your Supporting Information text/figures here. Please note: Supporting Display items, should be referred to as Figure S1, Equation S2, etc., in the main text…)

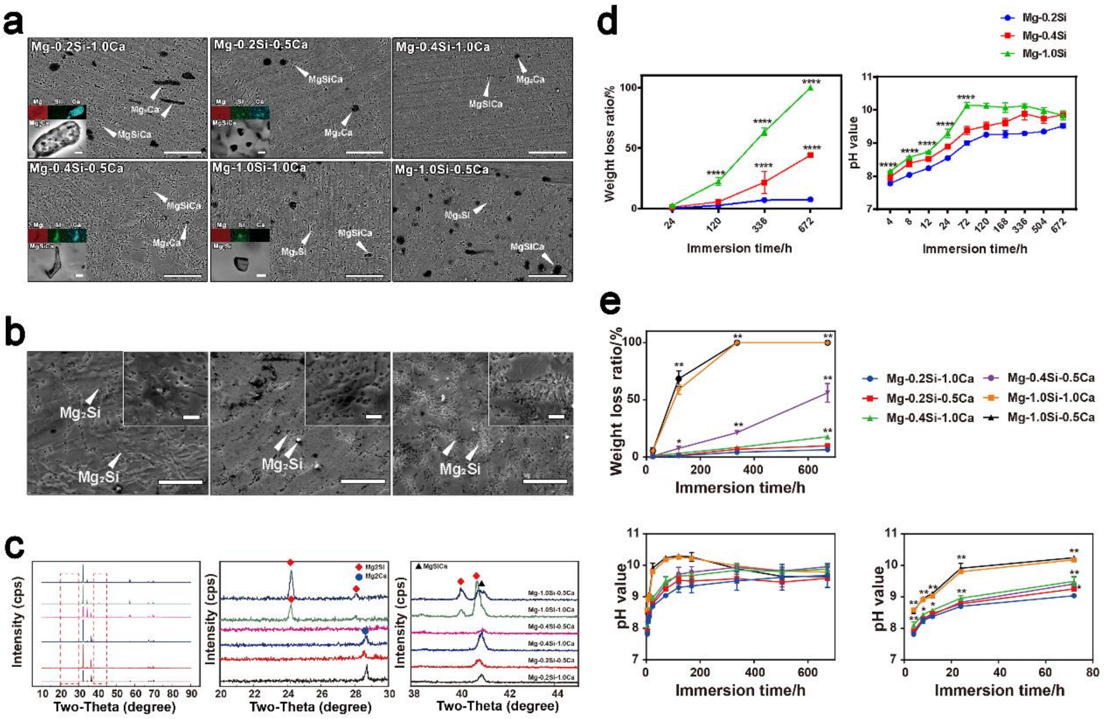

Supplementary Figure 1 (a, b) Microstructure of Mg-*x*(*x*=0.2, 0.4, 1.0)Si-*y*(*y*=0.5, 1.0)Ca alloys **(a)** and Mg-(0.2, 0.4, 1.0)Si alloys **(b)** observed by ESEM (scale bar = 30 μm) and higher magnification images of the intermetallic phase (scale bar = 1 μm) shown as inserts. **(c)** XRD spectra of Mg-*x*(*x*=0.2, 0.4, 1.0)Si-*y*(*y*=0.5, 1.0)Ca alloys. **(d, e)** The weight loss ratio and change of pH value of the solution of Mg-(0.2, 0.4, 1.0)Si alloys **(d)** and Mg-*x*(*x*=0.2, 0.4, 1.0)Si-*y*(*y*=0.5, 1.0)Ca alloys **(e)** during 28-day immersion in Hank's solution. These data are the mean ± s.d., n = 3 per group. * $P<0.05$ and ** $P<0.01$ by one-way ANOVA with Tukey's *post hoc* test.

a

Alloy	Corrosion Current Density (μA/cm²)	Corrosion rate (mm/y)	Corrosion Potential (V)
Mg-0.2Si	12.34±3.14	0.28±0.07	-1.47±0.01
Mg-0.4Si	16.68±5.01	0.38±0.11	-1.50±0.02
Mg-1.0Si	23.41±4.78	0.53±0.11	-1.43±0.01

b

Alloy	Corrosion current density (μA/cm²)	Corrosion rate (mm/y)	Corrosion potential (V)
Mg-0.2Si-1.0Ca	14.27±3.05	0.32±0.07	-1.46±0.04
Mg-0.2Si-0.5Ca	20.72±5.09	0.47±0.11	-1.48±0.03
Mg-0.4Si-1.0Ca	22.03±8.26	0.50±0.19	-1.46±0.02
Mg-0.4Si-0.5Ca	22.26±6.17	0.50±0.14	-1.51±0.05
Mg-1.0Si-1.0Ca	31.77±9.17	0.72±0.21	-1.45±0.02
Mg-1.0Si-0.5Ca	44.48±11.94	1.00±0.27	-1.46±0.03

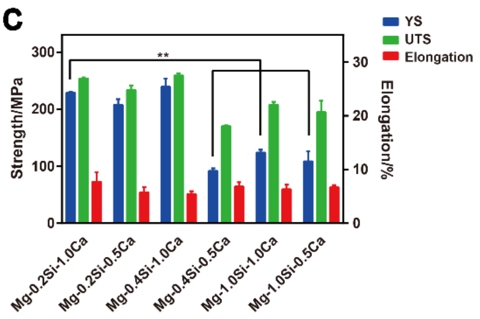

Supplementary Figure 2 (a, b) Corrosion current density, corrosion rate, and corrosion potential of Mg-(0.2, 0.4, 1.0)Si alloys **(a)** and Mg-*x*(*x*=0.2, 0.4, 1.0)Si-*y*(*y*=0.5, 1.0)Ca alloys **(b)** based on the results of electrochemical tests. **(c)** Yield strength, tensile strength, and elongation of Mg-*x*(*x*=0.2, 0.4, 1.0)Si-*y*(*y*=0.5, 1.0)Ca alloys. These data are the mean ± s.d., n = 3 per group. * $P<0.05$ and ** $P<0.01$ by one-way ANOVA with Tukey's *post hoc* test.

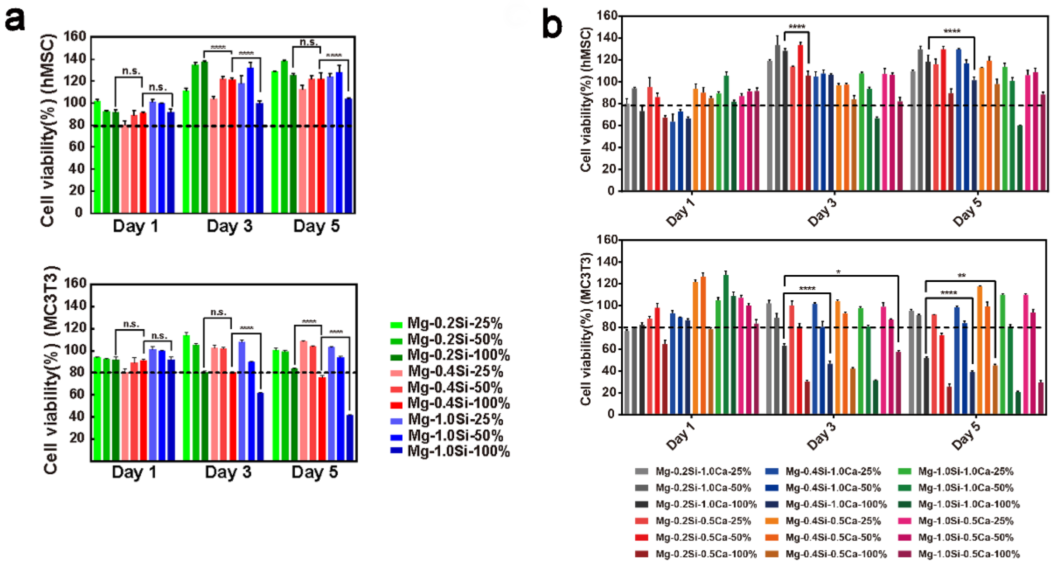

Supplementary Figure 3 (a, b) The cell viability of hMSC and MC3T3 cultured in the undiluted and diluted extracts of Mg-(0.2, 0.4, 1.0)Si alloys **(a)** and Mg-*x*(*x*=0.2, 0.4, 1.0)Si-*y*(*y*=0.5, 1.0)Ca alloys **(b)**. These data are the mean ± s.d., n = 3 per group. * $P<0.05$ and ** $P<0.01$ by one-way ANOVA with Tukey's *post hoc* test.

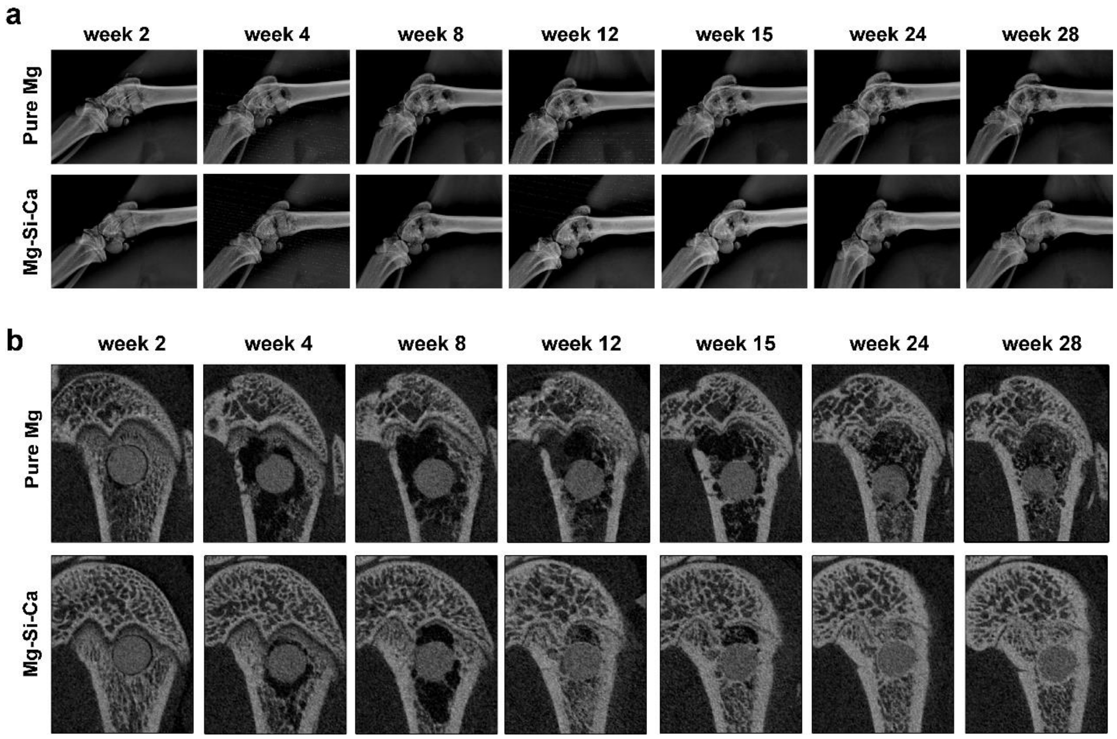

Supplementary Figure 4 (a) Representative radiographs of rats' femurs at postoperative weeks 2, 4, 8, 12, 15, 24, and 28. **(b)** Representative micro-CT images of rats' femurs at postoperative weeks 2, 4, 8, 12, 15, 24, and 28.

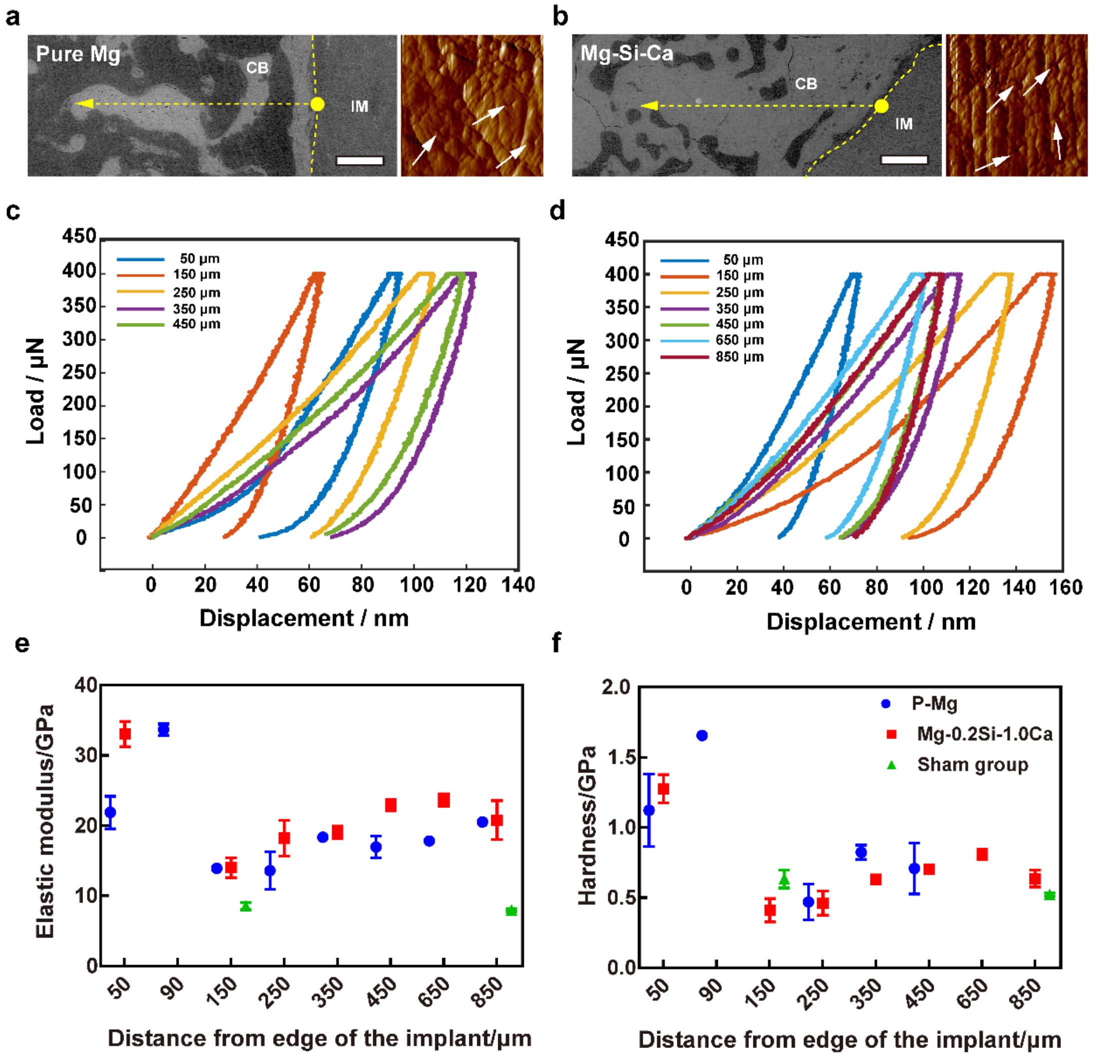

Supplementary Figure 5 ESEM images of the new bone formed around **(a)** pure Mg and **(b)** Mg-0.2Si-1.0Ca alloy. The dotted curve refers to the margin of the implant, and the nanoindentation test was conducted along the dotted line. Representative of the morphology of indentation on the right. Typical load-displacement curve (P-h curve) obtained during the nanoindentation of bone around **(c)** pure Mg and **(d)** Mg-0.2Si-1.0Ca alloy. **(e)** Elastic modulus and **(f)** hardness of the newly formed bone around pure Mg and Mg-0.2Si-1.0Ca alloys based on the nanoindentation tests. These data are the mean ± s.d., n = 5 per group.

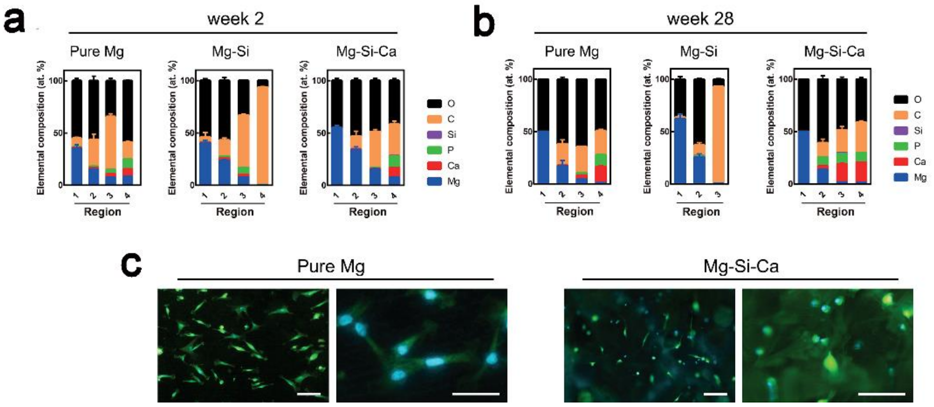

Supplementary Figure 6 (a, b) Chemical composition of multi-layered degradation products on implants at week 2 **(a)** and week 28 **(b)** after implantation determined using TEM-EDX. These data are the mean ± s.d., n = 5 per group. (**c**) Representative fluorescence images showing the attachment of hMSC on the surface of the implant 24 h after seeding (scale bar = 100 μm). The morphology of the attached hMSC is shown in high magnification on the right (scale bar = 50 μm).
